# Varicella Zoster Virus Induces Differential Cell-Type Specific Responses in Human Corneal Epithelial Cells and Keratocytes

**DOI:** 10.1167/iovs.18-25801

**Published:** 2019-02

**Authors:** Christina N. Como, Andrew N. Bubak, Anna M. Blackmon, Dallas Jones, Niklaus H. Mueller, Richard Davidson, Maria A. Nagel

**Affiliations:** 1 Department of Neurology, University of Colorado School of Medicine, Aurora, Colorado, United States; 2 Department of Ophthalmology, University of Colorado School of Medicine, Aurora, Colorado, United States; 3 Linda Crnic Institute for Down Syndrome, University of Colorado School of Medicine, Aurora, Colorado, United States

**Keywords:** varicella zoster virus, cornea, inflammation

## Abstract

**Purpose:**

While VZV DNA and antigen have been detected in acute and chronic VZV keratitis, it is unclear whether productive infection of corneal cells is ongoing or whether residual, noninfectious VZV antigens elicit inflammation. Herein, we examined VZV-infected primary human corneal epithelial cells (HCECs) and keratocytes (HKs) to elucidate the pathogenesis of VZV keratitis.

**Methods:**

HCECs and HKs were mock- or VZV infected. Seven days later, cells were examined for morphology, proinflammatory cytokine and matrix metalloproteinase (MMP) release, ability to recruit peripheral blood mononuclear cells (PBMCs) and neutrophils, and MMP substrate cleavage.

**Results:**

Both cell types synthesized infectious virus. VZV-infected HCECs proliferated, whereas VZV-infected HKs died. Compared to mock-infected cells, VZV-infected HCECs secreted significantly more IL-6, IL-8, IL-10, and IL-12p70 that were confirmed at the transcript level, and MMP-1 and MMP-9; conditioned supernatant attracted PBMCs and neutrophils and cleaved MMP substrates. In contrast, VZV-infected HKs suppressed cytokine secretion except for IL-8, which attracted neutrophils, and suppressed MMP release and substrate cleavage.

**Conclusions:**

Overall, VZV-infected HCECs recapitulate findings of VZV keratitis with respect to epithelial cell proliferation, pseudodendrite formation and creation of a proinflammatory environment, providing an *in vitro* model for VZV infection of corneal epithelial cells. Furthermore, the proliferation and persistence of VZV-infected HCECs suggest that these cells may serve as viral reservoirs if immune clearance is incomplete. Finally, the finding that VZV-infected HKs die and suppress most proinflammatory cytokines and MMPs may explain the widespread death of these cells with unchecked viral spread due to ineffective recruitment of PBMCs.

Varicella zoster virus (VZV) produces varicella then establishes latency in ganglionic neurons, including cranial nerve ganglia.^1^ With aging or immunosuppression, VZV reactivates and typically presents as zoster with 20% occurring in the ophthalmic distribution of the trigeminal nerve (herpes zoster ophthalmicus, HZO),^2^,^3^ of which 23% have eye involvement.^4^

Epithelial and stromal keratitis are corneal complications of VZV and can lead to vision loss after acute disease and during recurrence due to long-term corneal scarring and haze.^5^ While multiple studies have found viral DNA and/or antigen in early and late VZV keratitis, it is unclear whether these represent ongoing infection or persistent viral DNA and/or antigens that elicit a cell-mediated immune response. For example, punctate epithelial keratitis is transient early in disease, but may progress into pseudodendrites composed of swollen, heaped up, poorly adherent epithelial cells. VZV has been cultured from these abnormal epithelial cells, suggesting that active viral replication plays a role.^6^ Chronic epithelial pseudodendrites or mucus plaques can occur up to 2 years after HZO^6^–^11^ and contain VZV DNA.^10^,^12^ In contrast, VZV DNA has been detected in the cornea up to one month after acute epithelial keratitis and both VZV DNA and antigen have been detected in corneas up to 10 years after zoster without clinical symptoms and signs.^13^,^14^

Based on its rapid resolution upon corticosteroid treatment, stromal keratitis was thought to be an immune response to viral antigens within the corneal stroma, comprised of stromal fibroblasts underlying the epithelial cell layer.^15^,^16^ However, Matoba and colleagues^17^ found VZV DNA in the cornea of a patient with stromal keratitis, and a recent case of VZV keratitis demonstrated herpesvirus capsids in degenerative-appearing keratocytes, suggesting active VZV infection.^18^ Supporting evidence for active VZV infection in stromal keratitis is that in patients who develop recurrent keratitis, the persistence of VZV antigens seems unlikely because one would expect persistent inflammation; rather, the recurrent keratitis is more likely due to recurrent deposition of VZV to stroma with reactive inflammation.^19^ Thus, many questions remain concerning the role of active VZV infection in morphological changes seen in keratitis; the ability of corneal cells to support active VZV replication and transmit infectious virus to adjacent cells; the ability of VZV to persist in cornea, and; the role of VZV-induced inflammation in disease pathogenesis.

Since there is no animal model for VZV keratitis, we infected primary human corneal epithelial cells (HCECs) and stromal fibroblasts (keratocytes; HKs) with VZV and analyzed the cytopathology and inflammatory responses. Understanding the role of ongoing virus replication and inflammation in VZV keratitis can guide antiviral and corticosteroid therapy, mitigating ocular damage and vision loss produced by VZV.

## Materials and Methods

### Virus and Cells

The VZV Gilden strain (GenBank #MH379685) and primary HCECs and HKs from adult human cornea (ScienCell, Carlsbad, CA, USA) were used. Cells were confirmed by immunofluorescence antibody assay (IFA) and quantified by Fiji image processing software (https://fiji.sc/).

HCECs were seeded in corneal epithelial cell medium containing HCEC growth supplement/1% penicillin-streptomycin (20,000 cells/cm^2^; ScienCell). Cells were co-cultured with lysates from uninfected or VZV-infected human fetal lung fibroblast (HFL; ATCC, Manassas, VA) for 24 hours (200 plaque-forming units [PFU]/cm^2^),^20^ medium was then replaced to eliminate the possibility that ongoing infection was due to residual lysate. To confirm that no HFLs contaminated the corneal cultures, HFL lysates were plated and did not grow out cells. HKs were seeded in basal fibroblast medium containing 2% fetal bovine serum (FBS)/1% fibroblast growth serum/1% penicillin-streptomycin (ScienCell). After 24 hours, medium was changed to quiescent basal medium supplemented with 0.1% FBS/1% penicillin-streptomycin and replenished every 72 hours for 7 days. Quiescent HKs were mock- or VZV-infected with HFL lysates. Cells were counted at 1, 3, 5, and 7 days postinfection (DPI) at the height of cytopathic effect (CPE).

Human PBMCs (Stemcell Technologies, Vancouver, BC) and neutrophils purified from whole blood (Vitalant; Denver, CO, USA) using the direct human neutrophil isolation kit (EasySep; Stemcell, Cambridge, MA, USA) were used.

### Immunofluorescence Antibody Assay

At 7DPI, HCECs and HKs were analyzed by IFA using primary mouse anti-cytokeratin 18 (epithelial cell marker; 1:100), rabbit anti-fibronectin (fibroblast marker; 1:250), mouse anti-VZV glycoprotein B (gB; 1:250; Abcam, Cambridge, UK), and rabbit anti-GAPdH (Cell Signaling, Danvers, MA, USA) antibodies then secondary donkey anti-mouse AlexaFluor 594 IgG and donkey and anti-rabbit AlexaFluor 488 antibodies (1:500; Life Technologies, Grand Island, NY, USA) as described.^21^ Nuclei were stained with 2 μg/mL DAPI then visualized and analyzed by confocal microscopy.

### Transmission of VZV from Corneal Cells to HFLs

VZV-infected HCECs were harvested at 3, 5, and 7 DPI; each sample was serially diluted onto 70% confluent monolayers of uninfected HFLs in DMEM F12 medium with 10% FBS (ATCC) to demonstrate virus transmission from HCECs to HFLs. Similarly, VZV-infected HKs at 3, 5, and 7 DPI were diluted onto HFLs. Three days later, HFLs were stained with 0.1% crystal violet and PFUs/mL were calculated.

### Multiplex Electrochemiluminescence Immunoassay

Proinflammatory cytokines (IL-1β, IL-2, IL-4, IL-6, IL-8, IL-10, IL-12p70, IL-13, IFN-γ, and TNF-α) and MMPs in supernatants (MMP-1/2/3/9/10) were measured using commercial assays (Mesoscale Discovery, Rockville, MD, USA) as described.^21^

### RNA and Reverse Transcription-Quantitative Polymerase Chain Reaction (RT-qPCR)

RNA was extracted, cDNA transcribed then analyzed by RT-qPCR using primers to cytokines above and glyceraldehyde-3-phosphate-dehydrogenase (GAPdH, Integrated DNA Technologies, Coralville, IA, USA), as described.^21^,^22^ Data were normalized to GAPdH and analyzed using the ΔΔCt method or the ΔCt method if transcripts were detected in VZV-infected cells, but not in mock-infected cells.

### Immune Cell Migration Assay

At 7 DPI, mock- and VZV-infected supernatants were assayed for their ability to attract PBMCs and neutrophils. Supernatants were loaded in a 96-well chemotaxis plate (Neuroprobe, Gaithersburg, MD, USA), 5-μm pore size filters placed on top, immune cells pipetted onto filters (50,000 cells/well), and plate incubated at 37°C for 4 hours. Filters were removed; migrating immune cells were visualized by microscopy and quantified using ImageJ 3D Objects Counter (http://imagej.nih.gov/ij/; provided in the public domain by the National Institutes of Health, Bethesda, MD, USA). Positive control for PBMC and neutrophil migration was chemokine (C-C motif) ligand 2 (CCL2; Biolegend, San Diego, CA, USA) diluted in either HCEC or HK medium (20 pg/mL) and 50 ng/mL IL-8 (Biolegend), respectively. Negative controls for PBMC and migration assays were HCEC and HKs medium only. To determine specificity for IL8-induced neutrophil migration in infected supernatant, mouse monoclonal IL-8 blocking antibody was added at 1 μg/mL (αIL8, R&D Systems, Minneapolis, MN). Fold differences of VZV-infected cells and positive and negative controls were relative to mock-infected cells.

### MMP Activity

MMP general activity for mock- and VZV-infected HCEC and HK supernatants were quantified using a Fluorometric MMP Activity Assay (Abcam; Cambridge, UK). MMP-1 enzyme (500 pg/mL) served as positive control and MMP-1 enzyme plus MMP inhibitor (trypsin; 100 μg/mL) served as negative control (Anaspec; Fremont, CA, USA). Relative fluorescence units were measured using a fluorescent plate reader (excitation/emission: 485 nm/520 nm).

### Statistical Analysis

Statistical analysis was performed using graphing software (GraphPad Prism; GraphPad, San Diego, CA, USA). Cytokine and MMP differences among mock- and VZV-infected cells were determined by multiple unpaired t-tests with a False Discovery Rate (*q*-value ≤ 0.05) using the two-stage step-up method of Benjamini et al.^23^ Significant differences in cell count, viral titer, PBMC/neutrophil migration and MMP activity were determined using a 1-way ANOVA with a Tukey's multiple comparisons test. Alpha was set at 0.05 (*P < 0.05, **P < 0.01, ***P < 0.001).

## Results

### HCECs and HKs Are Permissive to VZV Infection and Transmit Virus

**Figure 1:**
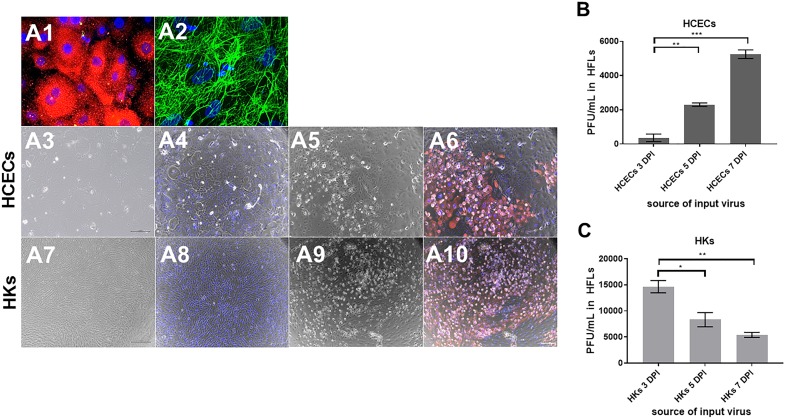
Phase-contrast imaging of VZV-infected primary HCECs and HKs. HCEC and HK cell types were verified by IFA. All DAPI-positive HCECs expressed the epithelial cell marker cytokeratin 18 (A1, red) and all DAPI-positive HKs expressed the fibroblast cell marker fibronectin (A2, green). HCECs and HKs were mock- or VZV-infected and analyzed at 7 days postinfection by phase microscopy and IFA using mouse anti-VZV glycoprotein E (gB) antibody. In mock-infected HCECs, phase images showed a cell monolayer without a CPE (A3) and no VZV gB (A4), whereas VZV-infected HCECs showed a CPE with areas of cell accumulation on phase-contrast (A5) that contained VZV gB by IFA (A6, red). In mock-infected HKs, phase images showed a monolayer of cells without CPE (A7) and no VZV gB (A8), whereas VZV-infected HKs showed a CPE on phase-contrast (A9) that corresponded to cells expressing VZV gB (A10, red). Blue color indicates cell nuclei. Mag 400X, A1 and A2; 100X, A3-A10. At 3, 5, and 7 days postinfection, infectious virus transmission from VZV-infected HCECs and HKs was measured by serially diluting cells onto uninfected HFLs. After 3 days of co-culture, HFLs were stained with crystal violet and the number of PFU/mL was determined. VZV-infected HCECs significantly increased the amount of PFU/mL at each time point: 3 DPI (367 ± 219), 5 DPI (2300 ± 82), 7 DPI (5250 ± 204; mean PFU/mL ± SEM; n = 3 [B]). In contrast, VZV-infected HKs significantly decreased PFU/mL at each time point: 3 DPI (14,666 ± 1171), 5 DPI (8333 ± 1353), 7 DPI (5400 ± 493; mean PFU/mL ± SEM; n = 3; [C]). Dashed lines represent a 1-fold (no) change relative to control groups (*P < 0.05, **P < 0.01, ***P < 0.001).

All DAPI-positive HCECs expressed cytokeratin 18 (total cells counted = 4328; Fig. 1A1, red); all DAPI-positive HKs expressed fibronectin (total cells counted = 3594; Fig. 1A2, green), indicating homogeneous cell cultures.

HCECs and HKs exposed to uninfected HFL lysates had no CPE or VZV gB (red; Figs. 1A3, 1A4 and 1A7, 1A8, respectively). HCECs exposed to VZV-infected HFL lysates had a CPE and contained regions of cells expressing VZV gB (Figs. 1A5, 1A6) that accumulated and spread, indicating that HCECs can harbor replicating VZV that spreads to adjacent cells. VZV-infected HKs demonstrated an expanding CPE with VZV gB expression (Figs. 1A9, 1A10).

Aside from VZV-infected corneal cells having the capacity to spread VZV to adjacent cells of the same type, VZV-infected HCECs and HKs were tested for their ability to transmit VZV to another cell type by cell-to-cell spread. VZV-infected HCECs and HKs at 3, 5, and 7 DPI were cocultured with uninfected HFLs; PFUs were counted at 3 DPI. The PFUs observed were due to VZV-infected HFLs since input VZV-infected HCECs perish in DMEM F12 medium, and input infected HKs were present in low amounts and are morphologically distinguishable. VZV-infected HKs die and form cell clearings, whereas VZV-infected HFLs swell and form syncytia. Input VZV-infected HCECs at 3, 5, and 7 DPI significantly increased the PFU/mL in HFLs at 367 ± 219, 2300 ± 82, and 5250 ± 204, respectively (Fig. 1B; mean ± SEM; *n* = 3), consistent with proliferating VZV-infected HCECs over time. In contrast, input VZV-infected HKs at 3, 5, and 7 DPI significantly decreased PFU/mL in HFLs at 14,666 ± 1171, 8333 ± 1353 and 5400 ± 493 (Fig. 1C; *n* = 3), consistent with dying VZV-infected HKs over time. Supernatant from infected cells were unable to transmit virus to HFLs demonstrating the absence of cell-free virus (data not shown).

### VZV-Infection Induced Cell Proliferation in HCECs and Cell Death in HKs

**Figure 2:**
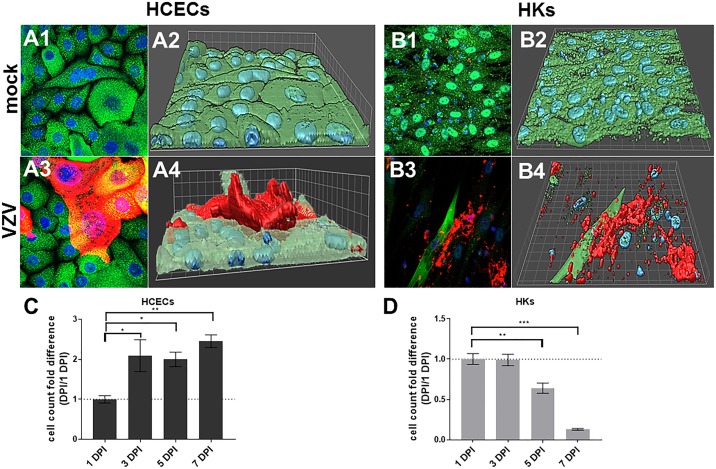
Differential morphology of VZV-infected primary HCECs and HKs. HCECs and HKs were mock- or VZV-infected and at 7 days postinfection, analyzed by IFA using mouse anti-VZV glycoprotein B (gB, red) and rabbit anti-GAPdH (green). Mock-infected HCECs expressed GAPdH but not VZV gB (A1); a corresponding surface plot showed HCECs in a monolayer (A2). VZV-infected HCECs contained infected cells expressing VZV gB (A3, red-yellow) and the corresponding surface plot showed that these cells were piling up over the monolayer (A4, red), similar to the elevated VZV-antigen-positive pseudodendrites seen in patients with VZV epithelial keratitis. Mock-infected HKs expressed GAPdH but not VZV gB (B1) and were present as a monolayer on a corresponding surface plot (B2). VZV-infected HKs contained classic plaques with areas of cell clearing/lysis and peripheral cells expressing VZV gB (B3) as confirmed on a surface plot (B4). Blue color indicates cell nuclei. Mag 400X. Cell counts of VZV-infected HCECs and HKs at 1, 3, 5 and 7 days postinfection were counted using a hemocytometer. Cell counts were normalized to 1 DPI and reported as a fold-difference. VZV-infected HCECs significantly increased cell counts at each time point: 3 DPI (2.09 ± 0.40), 5 DPI (2.00 ± 0.18), 7 DPI (2.45 ± 0.16; fold difference relative to 1 DPI ± SEM; n = 3 [C]), consistent with the lack of cell death seen on IFA images. VZV-infected HKs significantly decreased cell counts at 5 and 7 DPI compared to 1 DPI: 3 DPI (0.99 ± 0.07), 5 DPI (0.64 ± 0.07), 7 DPI (0.13 ± 0.01; fold difference relative to 1 DPI ± SEM; n = 3 [D]), consistent with cell death seen on IFA images. Dashed lines represent a 1-fold (no) change relative to control groups (*P < 0.05, **P < 0.01, ***P < 0.001).

Phase-contrast imaging indicated that VZV-infected HCECs proliferate; VZV-infected HKs sloughed off. Compared to mock- (Figs. 2A1, 2A2, surface plot), VZV-infected HCECs grew over the monolayer (Figs. 2A3, red, and 2A4, surface plot). Compared to mock- (Figs. 2B1, 2B2, surface plot), VZV-infected HKs showed cell clearing as infected cells died (Fig. 2B3, red, and 2B4, surface plot). VZV-infected HCECs significantly increased cell counts relative to 1 DPI at each time point: 3 DPI (2.09 ± 0.40), 5 DPI (2.00 ± 0.18), 7 DPI (2.45 ± 0.16; Fig. 2C; mean fold difference [MFD] ± SEM; *n* = 3). VZV-infected HKs significantly decreased cell counts at 5 and 7 DPI relative to 1 DPI: 3 DPI (0.99 ± 0.07), 5 DPI (0.64 ± 0.07), 7 DPI (0.13 ± 0.01; Fig. 2D; *n* = 3).

### Proinflammatory Cytokines and Infiltration of Immune Cells by VZV-Infected HCECs and HKs

**Figure 3:**
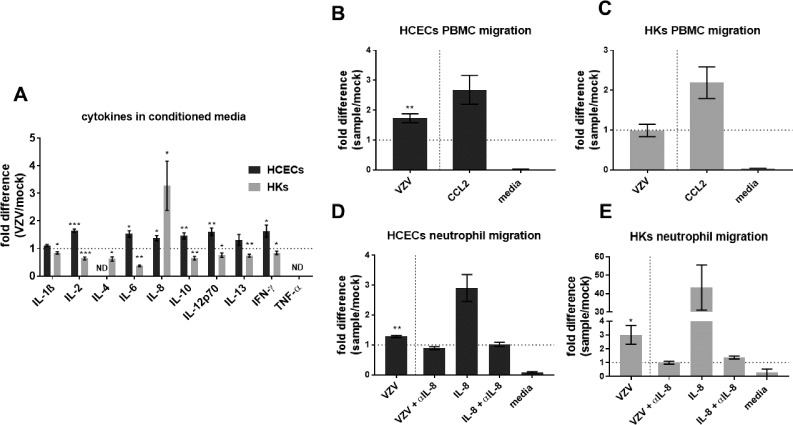
Proinflammatory cytokines and immune cell migration in conditioned supernatant from mock- and VZV-infected primary HCECs and HKs. At 7 days postinfection, conditioned supernatant from mock- and VZV-infected cells were analyzed for proinflammatory cytokines IL-1β, IL-2, IL-4, IL-6, IL-8, IL-10, IL-12p70, IL-13, IFN-γ, and TNF-α by multiplex assays (Meso Scale Discovery). Compared to the respective mock-infected cells, supernatant from VZV-infected HCECs contained significantly increased levels of IL-2, IL-6, IL-8, IL-10, IL-12p70 and IFNγ; IL-1β and IL-13 were unchanged and; IL-4 and TNF-α were not detected (ND; A, black bars; n = 5). Supernatant from VZV-infected HKs contained significantly decreased levels of IL-1β, IL-2, IL-4, IL-6, IL-10, IL-12p70, IL-13 and IFNγ; IL-8 was increased and; TNF-α was not detected (A, gray bars; n = 5). Results are reported as fold difference of VZV-infected compared to mock-infected cells. In chemotaxis assays, supernatants from mock- and VZV-infected cells at 7 days postinfection were placed in the bottom chamber, peripheral blood mononuclear cells (PBMCs) or neutrophils were placed in the top chamber separated from the bottom chamber by a 5-μm filter and the number of immune cells migrating through the filter and toward conditioned supernatant was quantitated 4 hours later. Compared to mock-infected supernatant, VZV-infected HCEC supernatant significantly increased PBMC infiltration (1.73 ± 0.15, mean fold difference ± SEM; n = 3), media only control had less migrating PBMCs than CCL2 (20 pg/mL, positive control) diluted in HCEC medium (0.03 ± 0.01 versus 2.68 ± 0.48, respectively; mean fold difference ± SEM; n = 3 [B]). Compared to mock-infected cells, VZV-infected HK supernatant did not increase PBMC migration (0.99 ± 0.16, mean fold difference ± SEM; n = 3), media only control had less migrating PBMCs than CCL2 diluted in HK medium (0.27 ± 0.27 versus 2.19 ± 0.40, respectively, mean fold difference ± SEM; n = 3 [C]). Compared to mock-infected HCEC supernatant, neutrophils significantly increased migration toward VZV-infected HCEC supernatant (1.29 ± 0.04, mean fold difference ± SEM; n = 3), anti-IL-8 antibody (αIL-8) in VZV-infected HCEC supernatant and an αIL-8 with IL-8 cytokine did not significantly increase neutrophil migration (0.89 ± 0.05 and 1.02 ± 0.07, respectively, mean fold difference relative to mock ± SEM; n = 3) and IL-8 diluted in HCEC medium significantly increased neutrophil infiltration (2.90 ± 0.45, mean fold difference ± SEM; n = 3 [D]). Compared to mock-infected HK supernatant, neutrophils significantly increased migration toward VZV-infected HK supernatant (3.01 ± 0.68, mean fold difference ± SEM; n = 3), αIL-8 in VZV-infected HK supernatant and a αIL-8 with IL-8 cytokine was not significantly increased (1.00 ± 0.10 versus 1.37 ± 0.11, respectively, mean fold difference relative to mock ± SEM; n = 3), IL-8 diluted in HK medium significantly increased PBMC infiltration (43.31 ± 12.22, mean fold difference ± SEM; n = 3 [E]). Dashed lines represent a 1-fold difference relative to control group (*P < 0.05, **P < 0.01, ***P < 0.001).

At 7 DPI, compared to mock-, VZV-infected HCEC supernatant significantly increased IL-2, IL-6, IL-8, IL-10, IL-12p70, and IFN-γ; no significant differences in IL-1β or IL-13 was seen and IL-4 and TNF-α were not detected (Table 1: mock- and VZV-HCEC mean concentrations, cytokine detection range and fold differences in columns 2 through 5, respectively; fold differences in Fig. 3A, black bars; MFD ± SEM; *n* = 5). To verify cytokine expression, transcripts were analyzed by RT-qPCR and fold differences between VZV- to mock-infected HCECs determined (Table 1, column 6). Compared to mock-, the following transcript changes corresponded to the secreted cytokines measured in VZV-infected HCECs: no significant increase in IL-1β; significant increases in IL-6, IL-8, IL-12p70, and; significant increase in IL-10 compared to no detection of IL-10 transcript in mock. The following fold differences in transcripts did not correspond to that detected for secreted proteins: IL-2, IL-4, IL-13, IFN-γ, and TNF-α.

**Table 1:** Meso Scale Discovery Analyte Levels and Transcript Levels from Mock- and VZV-Infected HCECs*

**Analyte**	**HCECs Mock, Mean ± SEM, pg/mL**	**HCECs VZV, Mean ± SEM, pg/mL**	**HCECs Fold Difference, VZV/Mock (*****P*****; *q*-value)**	**Detection Range LLOD-ULOD**	**HCECs Transcripts Fold Difference, ΔΔCt (*****P*** **Value)**
IL-1β	20.75 ± 6.03	22.94 ± 1.60	1.11 ± 0.03 Not significant	0.14–566	0.94 ± 0.19 (0.65)
IL-2	0.27 ± 0.05	0.44 ± 0.04	1.64 ± 0.06 (0.00006; 0.03)	0.34–1410	Undetectable
IL-4	0.03 ± 0.00	0.05 ± 0.04	Undetectable	0.06–246	2.59 ± 0.25 (0.008)
IL-6	15.73 ± 5	23.93 ± 4.12	1.52 ± 0.12 (0.03; 0.032)	0.17–712	1.45 ± 0.11 (0.04)
IL-8	861.19 ± 226.29	1181.6 ± 179.69	1.37 ± 0.09 (0.049; 0.037)	0.136–556	2.78 ± 0.33 (0.04)
IL-10	0.11 ± 0.02	0.17 ± 0.03	1.46 ± 0.11 (0.009; 0.016)	0.09–356	18.74 ± 0.03 (ΔCt) (0.00000004)
IL-12p70	0.15 ± 0.02	0.24 ± 0.04	1.60 ± 0.14 (0.008; 0.016)	0.12–497	3.09 ± 0.89 (0.049)
IL-13	3.86 ± 0.52	5.06 ± 1.68	1.31 ± 0.20 Not significant	0.12–492	27.63 ± 2.08 (0.009)
IFN-γ	1.01 ± 0.06	1.65 ± 0.49	1.63 ± 0.22 (0.038; 0.033)	0.38–1380	Undetectable
TNF-α	Undetectable	Undetectable	Undetectable	0.09–369	4.93 ± 0.29 (0.03)

LLOD, lower limits of detection ± 2.5 standard deviation; ULOD, upper limits of detection ± 2.5 standard deviation.

* Data was analyzed by an unpaired *t*-test corrected; fold difference (VZV/mock) ± SEM.

At 7 DPI, compared to mock-, VZV-infected HK supernatant had significantly decreased concentrations of IL-1β, IL-2, IL-4, IL-6, IL-10, IL-12p70, IL-13, and IFN-γ; significantly increased levels of IL-8 and; no detection of TNF-α (Table 2: mock- and VZV-infected HK mean concentrations, cytokine detection range and fold differences in columns 2 through 5, respectively; fold differences in Fig. 3A, gray bars; MFD ± SEM; *n* = 5). To verify cytokine expression, transcripts from mock- and VZV-infected HKs were analyzed by RT-qPCR and fold differences between VZV- to mock-infected HKs determined (Table 2, column 6). Compared to mock-, IL-8 transcript was significantly increased and TNF-α transcript was not detected corresponding to their secreted cytokine levels during VZV infection. The following fold differences in transcripts did not correspond to that detected for secreted proteins: IL-1β, IL-2, IL-4, IL-6, IL-12p70, IL-10, IL-13 and IFN-γ.

**Table 2:** Meso Scale Discovery Analyte Levels from Mock- and VZV-Infected HKs

**Analyte**	**HKs Mock, Mean ± SEM, pg/mL**	**HKs VZV, Mean ± SEM, pg/mL**	**HKs Fold Difference, VZV/Mock (*****P*****; *q*-value)**	**Detection Range LLOD-ULOD**	**HKs Transcripts Fold Difference, ΔΔCt (*****P*** **value)**
IL-1β	1.79 ± 0.20	1.50 ± 0.17	0.84 ± 0.04 (0.041; 0.006)	0.14–566	Undetectable
IL-2	1.00 ± 0.11	0.64 ± 0.12	0.64 ± 0.05 (0.0009; 0.0009)	0.34–1410	Undetectable
IL-4	0.08 ± 0.02	0.05 ± 0.01	0.62 ± 0.08 (0.036; 0.006)	0.06–246	8.14 ±1.41 (0.009)
IL-6	9.38 ± 3.42	3.49 ± 0.70	0.37 ± 0.03 (0.005; 0.002)	0.17–712	24.40 ± 5.58 (0.014)
IL-8	27.61 ± 7.58	90.48 ± 24.59	3.27 ± 0.69 (0.07; 0.008)	0.136–556	38.37 ± 30.02 (0.04)
IL-10	0.47 ± 0.04	0.30 ± 0.07	0.65 ± 0.07 (0.002; 0.001)	0.09–356	Undetectable
IL-12p70	0.49 ± 0.05	0.37 ± 0.09	0.76 ± 0.08 (0.037; 0.006)	0.12–497	1.88 ± 0.70 (0.02)
IL-13	11.31 ± 1.33	8.35 ± 1.39	0.74 ± 0.05 (0.009; 0.002)	0.12–492	27.63 ± 2.08 (0.0003)
IFN-γ	2.62 ± 0.07	2.20 ± 0.38	0.84 ± 0.06 (0.04; 0.006)	0.38–1380	Undetectable
TNF-α	Undetectable	Undetectable	Undetectable	0.09–369	Undetectable

Data was analyzed by an unpaired *t*-test corrected; fold difference (VZV/mock) ± SEM.

Relative to mock-, VZV-infected HCEC supernatant significantly increased PBMC infiltration (1.73 ± 0.15), consistent with the elevated proinflammatory cytokine levels detected. Media only control had less migrating PBMCs than CCL2 diluted in HCEC medium (0.03 ± 0.01 vs. 2.68 ± 0.48, respectively; Fig. 3B; MFD ± SEM; *n* = 3). In contrast, VZV-infected HK supernatant did not increase PBMC migration (0.99 ± 0.16), consistent with the suppression of proinflammatory cytokines. Media only control had less migrating PBMCs than CCL2 diluted in HK medium (0.27 ± 0.27 vs. 2.19 ± 0.40, respectively; Fig. 3C; MFD ± SEM; *n* = 3).

Relative to mock-, VZV-infected HCEC supernatant significantly increased neutrophil migration (1.29 ± 0.04). Anti-IL-8 in VZV-infected supernatant, αIL-8 with IL-8 cytokine diluted in media and media only did not significantly increase migration (0.89 ± 0.05, 1.02 ± 0.07, 0.08 ± 0.02, respectively). IL-8 cytokine diluted in HCEC medium significantly increased neutrophil infiltration (2.9 ± 0.45; Fig. 3D; MFD ± SEM; *n* = 3).

Compared to mock-, VZV-infected HK supernatant significantly increased neutrophil migration (3.01 ± 0.68), consistent with the elevated levels of IL-8 (a neutrophil chemoattractant). Anti-IL-8 in VZV-infected HK supernatant, αIL-8 with IL-8 cytokine in media and media only did not significantly increase migration (1.00 ± 0.1, 1.37 ± 0.11, 0.27 ± 0.27, respectively). IL-8 diluted in HK medium significantly increased PBMC infiltration (43.31 ± 12.22; Fig. 3E; MFD ± SEM; *n* = 3). The ability of αIL-8 to block migration of neutrophils indicates that IL-8 in the infected conditioned media was responsible for neutrophil chemotaxis.

### MMP Secretion and Activity in VZV-Infected HCECs and HKs

**Figure 4:**
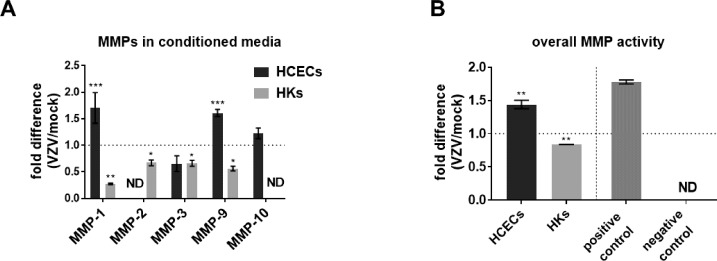
Matrix metalloproteinases (MMPs) and activity in conditioned supernatant from mock- and VZV-infected primary HCECs and keratocytes (HKs). At 7 days postinfection, mock- and VZV-infected conditioned supernatant from HCECs and HKs were analyzed for MMPs-1, -2, -3, -9 and -10 using Meso Scale Discovery multiplex assays. Compared to the respective mock-infected cells, conditioned supernatant from VZV-infected HCECs significantly increased levels of MMP-1 and -9, but not MMP-3 or -10; MMP-2 was not detected (ND; A, black bars), whereas supernatant from VZV-infected HKs contained significantly decreased levels of MMP-1, -2, -3 and -9; MMP-10 was ND (A, gray bars). Overall MMP activity was measured as substrate cleavage, reported as relative fluorescence unit (RFU, excitation 485 nm and emission 520 nm). Compared to the respective mock-infected cells, supernatant from VZV-infected HCECs had significantly increased MMP activity (1.44 ± 0.06, mean fold difference ± SEM; n = 3), while supernatant from VZV-infected HKs had significantly decreased MMP activity (0.84 ± 0.00, mean fold difference ± SEM; n = 3 [B]). MMP-1 enzyme (500 pg/mL) served as the positive control (1.78 ± 0.03, mean fold difference ± SEM; n = 3), while MMP-1 enzyme with the trypsin deactivating enzyme at 100 μg/mL (0.01 ± 0.00, mean fold difference ± SEM; n = 3) was used as the negative control. Dashed lines represent a 1-fold difference relative to control group (*P < 0.05, **P < 0.01, ***P < 0.001).

Compared to mock-, VZV-infected HCEC supernatant significantly increased MMP-1 (1.70 ± 0.29) and MMP-9 (1.61 ± 0.07), but not MMP-3 or MMP-10; MMP-2 was not detected (Fig. 4A, black bars; MFD ± SEM; *n* = 3). In contrast, VZV-infected HK supernatant significantly decreased MMP-1 (0.28 ± 0.01), MMP-2 (0.67 ±, 0.05), MMP-3 (0.66 ± 0.06) and MMP-9 (0.56 ± 0.04); MMP-10 was not detected (Fig. 4A, gray bars; MFD ± SEM; *n* = 3).

Compared to mock-, VZV-infected HCECs significantly increased MMP activity (1.44 ± 0.06), whereas VZV-infected HKs significantly decreased MMP activity (0.84 ± 0.0009; Fig. 4B; MFD ± SEM; *n* = 3). Positive control MMP-1 enzyme had increased activity (1.78 ± 0.03) compared to the negative control that was not detected.

## Discussion

Herein, we show both HCECs and HKs can harbor productive VZV infection yet differ with respect to cytopathology, proinflammatory cytokine production and matrix metalloproteinase release, which may contribute to the clinical differences in VZV epithelial and stromal keratitis.

The question of whether HKs are able to synthesize infectious virions was recently raised following a case report of a patient who developed VZV stromal keratitis after zoster vaccination.^18^ VZV particles appeared inside keratocytes; however, capsids were empty and it was unclear whether VZV was able to complete all the replication steps and release complete virions to infect adjacent keratocytes.^19^ To our knowledge, only one previous study examined the effects of VZV infection on human corneal stromal fibroblasts in vitro,^24^ which showed that VZV can infect stromal fibroblasts and that VZV deficient in functional ORF66 protein kinase expression is severely growth-impaired in these cells. Our study showed that both VZV-infected HCECs and HKs can synthesize infectious virus particles following exposure to VZV-infected HFL lysates, as demonstrated by the proliferation and “piling up” of HCECs expressing VZV glycoproteins, reminiscent of epithelial pseudodendrites, and by an expanding cytopathic effect in VZV-infected HKs that express VZV glycoproteins at the periphery of plaques, reminiscent of degenerating stromal cells seen by Jastrzebski and colleagues.^18^ While conditioned supernatant from VZV-infected HCECs and HKs were unable to infect HFLs, indicating that cell-free virus was not produced, infected HCECs and HKs were able to transmit infectious virus particles to HFLs by cell-to-cell spread, demonstrating that HCECs and HKs are permissive to VZV infection.

Compared to mock-, VZV-infected HCECs secreted significantly higher levels of IL-2, IL-6, IL-8, IL-10, IL-12p70 and IFN-γ in the conditioned medium; no significant differences in IL-1β or IL-13 were measured. IL-4 and TNF-α were not detected. However, only IL-1β, IL-6, IL-8, IL-10, and IL-12p70 were confirmed at the transcript level. The reason(s) for the disparities in transcript and secreted cytokine levels remain to be determined. Consistent with the increased proinflammatory cytokine levels, VZV-infected HCEC supernatant recruited PBMCs and neutrophils in a migration assay, consistent with inflammatory infiltrates observed in VZV-infected corneal epithelium of patients. The number of proinflammatory cytokines secreted by VZV-infected HCECs is greater than that previously reported in VZV-infected human brain vascular adventitial fibroblasts (HBVAFs), vascular smooth muscle cells (HBVSMCs), perineurial cells (HPNCs) and HFLs.^25^ During VZV infection, IL-8 was increased in all four of these primary human cell cultures; IL-6 was increased in all but HBVSMCs, while IL-2 was increased only in HPNCs. However, there were no increases in IL-4, IL-10, IL-12p70 or IFN-γ, possibly due to the different VZV strains used (VZV Ellen strain in the previous report and the Gilden strain herein) or differential cell type specific responses to infection. The cytokines secreted from VZV-infected HCECs differ from those secreted from herpes simplex-1 (HSV-1; KOS strain)-infected HCECs. While both viruses led to an increase in IL-6 and IL-8, HSV-1–infected HCECs also showed an increase in TNF-α and IFN-β,^26^ which was not detected and not measured in VZV-infected HCECs, respectively. In a study of HSV-1-infected human cornea organotypic cultures, IL-6 and IL-1β were not significantly increased, but TNF-α was modestly increased; IL-17 was not detected.^27^ These differential cytokine responses in HCECs against two different viruses may contribute to differences in the level of inflammation and morphological changes (i.e., ulcerations and perforation, seen in VZV and HSV-1 epithelial keratitis).

In VZV-infected HCECs, levels of secreted MMP-1 and MMP-9 were significantly increased, unlike the HSV-1-induced increases in MMP-2 and MMP-9 in HCECs.^28^ MMP-1 is an enzyme that cleaves collagen that is present in the extracellular matrix of the cornea.^29^ Similarly, MMP-9 degrades denatured collagen.^30^ Both MMPs can mediate epithelial-stromal interactions, immune cell infiltration, corneal damage and corneal ulceration.^31^ Since the ability of MMPs to cleave extracellular matrix depends on the balance of activated MMPs and tissue inhibitors of MMPs, an MMP activity assay was used that showed increased extracellular matrix substrate cleavage. This increase in MMP activity associated with VZV infection of HCECs could lead to the loss of epithelial layer integrity and promote an environment that is more permissive to immune cell infiltration and epithelial cell migration and proliferation.

Compared to mock-infected HKs, VZV-infected HKs only secreted significantly higher levels of IL-8 that was confirmed at the transcript level, similar to HSV-1-induced IL-8 secretion in keratocytes;^32^ conditioned supernatant recruited neutrophils in the migration assay. Specificity for increased IL-8 in HK supernatant acting as a neutrophil attractant is seen by decreased infiltration when IL-8 was neutralized with an anti-IL-8 antibody. In addition, MMP-1, -2, -3, and -9 were suppressed in VZV-infected HKs. The inability of VZV-infected HKs to secrete proinflammatory cytokines, recruit PBMCs and cleave extracellular matrix, in contrast to the ability of VZV-infected HCECs to create a proinflammatory environment, may be due to differences in developmental origins: HCECs are embryonic ectoderm-derived, whereas HKs are neural crest-derived.^33^

Overall, our data shows that VZV-infected HCECs recapitulate the clinical findings seen in patients with VZV keratitis, including epithelial cell proliferation, pseudodendrite formation and increased proinflammatory cytokines and MMPs, providing an in vitro model for VZV infection of HCECs. The proliferation and persistence of VZV-infected HCECs suggest that these cells may serve as viral reservoirs if immune clearance is incomplete. Finally, the VZV-induced death of HKs and suppression of most proinflammatory cytokines and MMPs may explain the widespread death of these cells with unchecked viral spread due to ineffective recruitment of PBMCs.
